# Inhibitory effect of blocking TGF-β/Smad signal on injury-induced fibrosis of corneal endothelium

**Published:** 2008-12-11

**Authors:** Takayoshi Sumioka, Kazuo Ikeda, Yuka Okada, Osamu Yamanaka, Ai Kitano, Shizuya Saika

**Affiliations:** 1 Department of Ophthalmology, Wakayama Medical University, Wakayama, Japan; 2 Department of Anatomy, Graduate School of Medicine, Osaka City University, Osaka, Japan

## Abstract

**Purpose:**

To understand the role of TGF-β related signals in the repair of a corneal endothelium defect and also to evaluate the therapeutic effect of *Smad7* gene transfer on injury induced fibrosis of the corneal endothelium in rats.

**Methods:**

(1) Japanese albino rabbits (n=108) were used. Blocks of central cornea (4×4 mm) were prepared. After partially scraping the endothelium to produce a defect, the blocks were organ cultured for 24 h in the presence of either exogenous growth factors, transforming growth factor β (TGF-β)-neutralizing antibody, or inhibitors of each TGF-β related signal. Endothelium repair was assayed under light microscopy. (2) Adult Wistar rats (n=62) were then used. Smad7 expressing adenoviral vector (Smad7-Ad) or non-functioning control vector (Cre-Ad) was administered to the anterior chamber of an eye. The cornea was burned with topical 1 N NaOH (10 μl) three days later. After specific intervals, the eye was histologically observed.

**Results:**

(1) The endothelial layer that elongated toward the defect lacked proliferation after 24 h in organ culture. Endogenous TGF-β was required for endothelium defect repair. Inhibition of p38 and Erk but not c-Jun NH_2_-terminal kinase (JNK) and ALK5 signal (Smad) retarded such cell spreading. (2) Adenoviral Smad7 overexpression suppressed fibrogenic reaction of the endothelium of an alkali-burned cornea as evaluated by immunohistochemistry for phospho-Smad2, collagen I, and α-smooth muscle actin, a marker for endothelial-mesenchymal transition (EnMT), and by electron microscopy.

**Conclusions:**

Inhibition of Smad and JNK signals do not affect corneal endothelium defect repair. Inhibition of Smad suppresses fibrogenic reaction via EnMT of corneal endothelium in vivo.

## Introduction

A healthy endothelium is essential for the maintenance of corneal homeostasis and transparency of the cornea. Defects in the endothelium are repaired mainly by cell size enlargement and cell migration in humans, and additional cell proliferation also participates in such repair in rodents.

An alkali burn in the cornea is a clinically serious condition because it damages not only the epithelium and stroma but also the endothelium. During healing after an alkali burn, the fibrous structure is formed in the endothelial layer beneath Descemet’s membrane [1-3]. Formation of such fibrous structure impairs the physiologic function of the endothelium to maintain transparency. In the process of fibrogenic reaction, corneal endothelial cells undergo epithelial/endothelial mesenchymal transition (EMT/EnMT) and transform to fibrogenic myofibroblasts [4-7]. EMT serves as the pathogenesis of fibrotic diseases in many tissues such as the eye lens, retinal pigment epithelium, kidney, liver, and lungs [8-12]. EMT is modulated by a set of various growth factors/cytokines. Among them, it is believed that transforming growth factor β (TGF-β) is one of the most potent growth factors involved in myofibroblast generation through EMT [13-15]. Indeed, in many tissues, blocking TGF-β signaling by targeted deletion of *Smad3* or gene introduction of *Smad7*, an inhibitory Smad, reportedly prevents EMT and pathological tissue fibrosis. Thus, blocking TGF-β signaling or introducing *Smad7* is of therapeutic value [16-19]. However, it is not fully examined if an interfering TGF-β signal modulates EMT of corneal endothelial cells and also exhibits a therapeutic effect.

TGF-β activates not only Smad signals but also other cytokines/growth factors such as mitogen-activated protein kinase (MAPK), p38MAPK, and c-Jun NH_2_-terminal kinase (JNK) [20-22]. Because migration is a major component of wound healing in the corneal endothelium, strategies of inhibition of unfavorable EMT of the corneal endothelium is not to be accompanied with an impairment of cell migration.

In the present study, we first examined which TGF-β related cytoplasmic signaling is essential for the repair of a defect in the corneal endothelium in organ culture, and then we investigated if a *Smad7* gene transfer exhibits a therapeutic effect on injury induced fibrogenic reaction of the corneal endothelium. It is required to know the role of each TGF-β related signal in endothelial cell repair to avoid inhibition of the cell migration promoting signal when we try to block unfavorable EnMT by targeting TGF-β related signal(s).

## Methods

Experiments were approved by the DNA Recombination Experiment Committee and the Animal Care and Use Committee of Wakayama Medical University (Wakayama, Japan) and were conducted in accordance with the Association for Research in Vision and Ophthalmology Statement for the Use of Animals in Ophthalmic and Vision Research.

### Migration of corneal endothelial cells in organ culture

**Figure 1:**
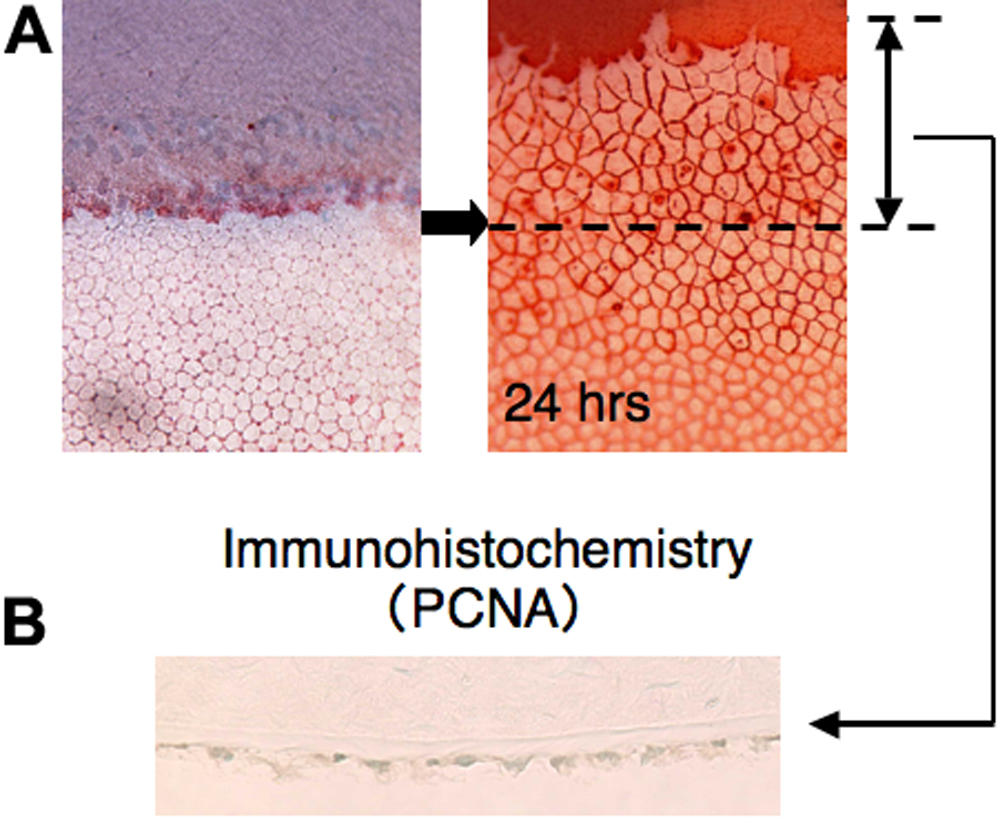
Method for assaying spreading of the endothelial cell sheet in organ culture. A: After 24 h culture, the length of the endothelium path on the denuded area was measured. B: The spreading sheet of the endothelial cells lacks proliferating cell nuclear antigen (PCNA) at 24 h in the control culture.

First, Japanese albino rabbits (n=108) were used. After sacrificing, the central cornea was excised. Blocks of the cornea (4 mm×4 mm) were prepared. The endothelium was partially (approximately 50%) removed by scraping with a glass coverslip as shown in Figure 1A. The endothelium of the corneal periphery was preserved. The cornea block with a endothelial defect was then organ cultured for 24 h in serum-free Dulbecco’s modified Eagle medium supplemented with antibiotics and an antimycotic in the presence or absence of each reagent. Reagents added to the medium were recombinant human epidermal growth factor (EGF, 10.0 ng/ml; R&D systems, Minneapolis, MN), human TGF-β1 (1.0 ng/ml; R&D systems), human TGF-β2 (1.0 ng/ml; R&D systems), EGF (10.0 ng/ml) plus TGF-β1 (1.0 ng/ml), EGF (10.0 ng/ml) plus TGF-β2 (1.0 ng/ml), a p38MAPK inhibitor called SB203580 (10 μM; Sigma-Aldrich, St Louis, MO), a MAPK inhibitor called U0126 (10 μM; Calbiochem, San Diego, CA), a JNK inhibitor (5 μM; Calbiochem), and a ALK5 inhibitor called SB431542 (10 μM; Sigma-Aldrich). After being cultured for 24 h, the corneal blocks were stained with 0.2% Alizarin red and 0.25% trypan blue, and the endothelium was observed under light microscopy. The distance between the line of the original defect margin and the spreading tip of the newly migrating endothelium was measured and served as the endothelium migration. Immunohistochemistry for proliferating nuclear cell antigen (PCNA) was performed to examine if the cell spreading (or healing) of the endothelium was associated with cell proliferation during the 24 h period (Figure 1B). Specificity of the inhibitory effect of SB431542 on Smad2/3 signaling was established [23]. To examine the role of endogenous TGF-β in endothelium spreading, the medium was supplemented with either mouse monoclonal anti-human TGF-β neutralizing antibody (20 μg/ml, R&D systems) or mouse IgG1 derived from BALB/c strain (20 μg/ml, Sigma-Aldrich).

### *Smad7* gene transfer using a Cre/LoxP adenoviral vector

Here, adult Wistar rats (n=4) were used. Efficacy of gene transfer by this system was initiated by using adenoviral vectors carrying CAG (cytomegalovirus enhancer, chicken β-actin promoter plus a part of the 3′ untranslated region of rabbit β-globin) promoter driven Cre recombinase (Cre-Ad) and LoxP-neomycin resistance gene-LoxP (LNL)-green fluorescein protein (GFP) expressing vector. The cryosection (7 μm thick) of the treated cornea was prepared and observed under fluorescent microscopy as previously reported [24].

Adult Wistar rats (n=12) were again used for the adenoviral gene transfer. Adenoviral gene transfer of mouse *Smad7* cDNA was performed by using the Cre/LoxP system of the adenoviral vector (Takara, Tokyo, Japan) as previously reported by us [24]. The viral vector (2.0×10^4^ PFU/ml, either Cre-Ad alone or a mixture of Cre-Ad and LNL-Smad7-expressing vector [Smad7-Ad]) was mixed with 2.0 μl of hyaluronan (OPEGAN®, Santen Pharmaceutical, Osaka, Japan) and injected into the anterior chamber of one eye in each rat. After three days, one week, and two weeks, the animals were killed at each time period and the corneas were excised. Cre recombinase expressed by Cre-Ad deletes the LoxP site of the stuffer in the promoter region of the LNL-Smad7-Ad, and thus, *Smad7* mRNA is expressed. Total RNA was extracted by using Sigma GenEluted^TM^ Mammalian Total RNA Miniprep Kit (Sigma-Aldrich) and processed for real-time reverse transcription polymerase chain reaction (RT–PCR) for *Smad7* mRNA as previously reported by us [24]. The cornea was also processed for immunohistochemistry for Smad7 as previously reported [24].

### Cornea alkali burn model of corneal endothelium fibrosis

Adult Wistar rats (n=46) were used here as well. The viral vector (either Cre-Ad or Smad7-Ad) in hyaluronan was injected into the anterior chamber of an eye of each rat. After three days, 10 μl of 1.0 N NaOH was topically applied to the cornea, and ofloxacin ointment was administered to reduce the risk of bacterial contamination. After three days, one week, and two weeks, the animals were killed at each time period by an overdose of pentobarbital, i. p., and the eye was enucleated, fixed with 4% paraformaldehyde, and embedded in paraffin (n=38) as previously reported [24]. Deparaffinized sections (5 µm thick) were processed for immunohistochemistry for phospho-Smad2, α-smooth muscle actin (myofibroblast marker), type I collagen, and PCNA. Eight corneas of each group at week 1 post-alkali burn were fixed in 2% glutaraldehyde in 0.1 M phosphate buffer. The samples were dehydrated though a graded series of ethanol and a critical point dryer. After coating with gold by using ion spatter, the endothelium was observed under scanning electron microscopy.

## Results

### Endothelium migration and cytokine signaling

**Figure 2:**
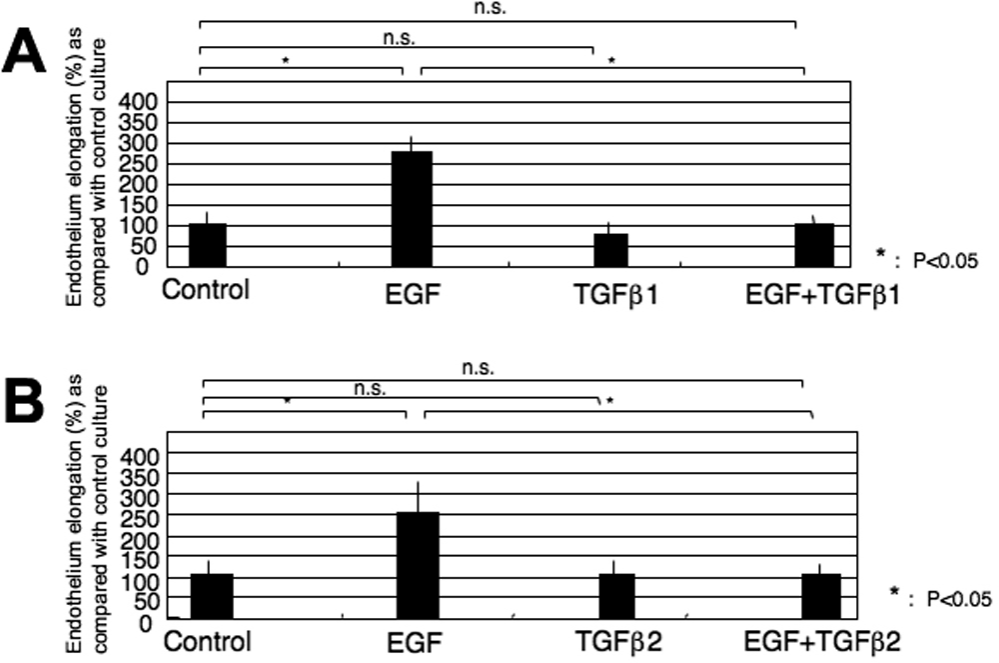
Effects of exogenous EGF, TGF-β1, or TGF-β2 on the spreading of the corneal endothelium in organ culture. Exogenous EGF promoted endothelium spreading. While adding exogenous TGF-β1 (A) or TGF-β2 (B) to the medium exhibited no effect on endothelium sheet elongation, it counteracted the promotion of endothelial sheet elongation by exogenous EGF.

After a 24 h incubation, the endothelium defected corneal block lacked PCNA labeled endothelium (Figure 1B), and thus, later elongation of the endothelium toward the defect was performed by cell migration during the first 24 h in organ culture. Exogenous EGF promoted endothelium repair. While adding exogenous TGF-β1 or TGF-β2 to the medium exhibited no effect on endothelium sheet spreading, it counteracted the promotion of the endothelial sheet spreading by exogenous EGF (Figure 2).

**Figure 3:**
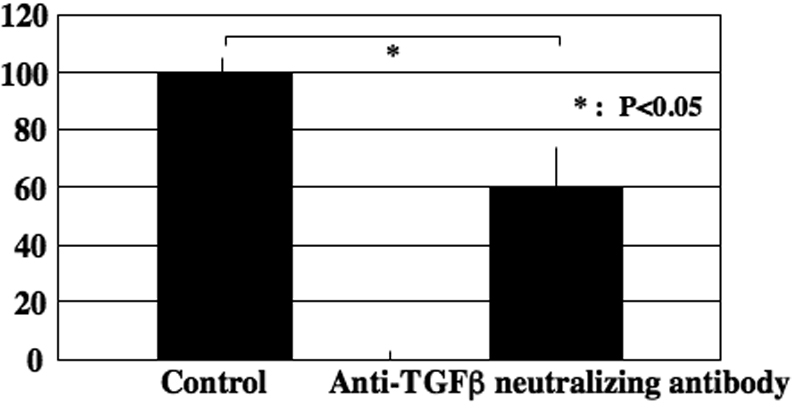
The role of endogenous TGF-β was evaluated by using neutralizing antibody. Adding neutralizing anti-TGF-β antibody retarded endothelium repair.

**Figure 4:**
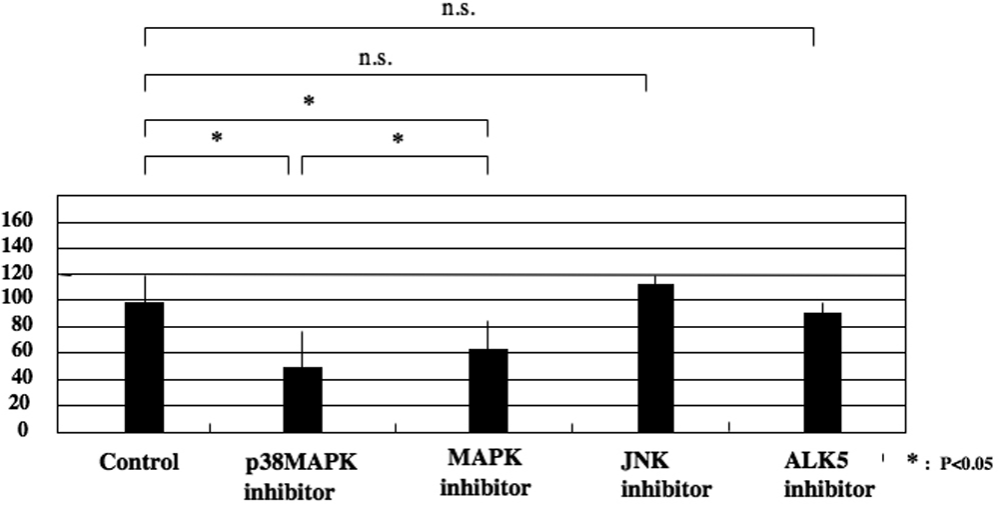
Roles of TGF-β related signaling on the spreading of the corneal endothelium in organ culture. The p38 inhibitor, SB203580, and the MAPK inhibitor, U0126, hindered endothelium sheet elongation (inhibition: SB203580>U0126). Neither the JNK inhibitor nor the ALK5 inhibitor, SB431542, (blocking Smad2/3 signal) have a significant effect on its repair.

The role of endogenous TGF-β was evaluated by using a neutralizing antibody. Adding the neutralizing anti-TGF-β antibody retarded endothelium repair (Figure 3). As for the roles of TGF-β related signaling, specific inhibitors were used. The p38 inhibitor, SB203580, and the MAPK inhibitor, U0126, hindered endothelium sheet spreading (inhibition: SB203580>U0126; Figure 4). Neither the JNK inhibitor nor the ALK5 inhibitor, SB431542, (thus blocking the Smad2/3 signal) had a significant effect on its repair (Figure 4).

### *Smad7* gene transfer by using Cre/LoxP adenoviral vector

**Figure 5:**
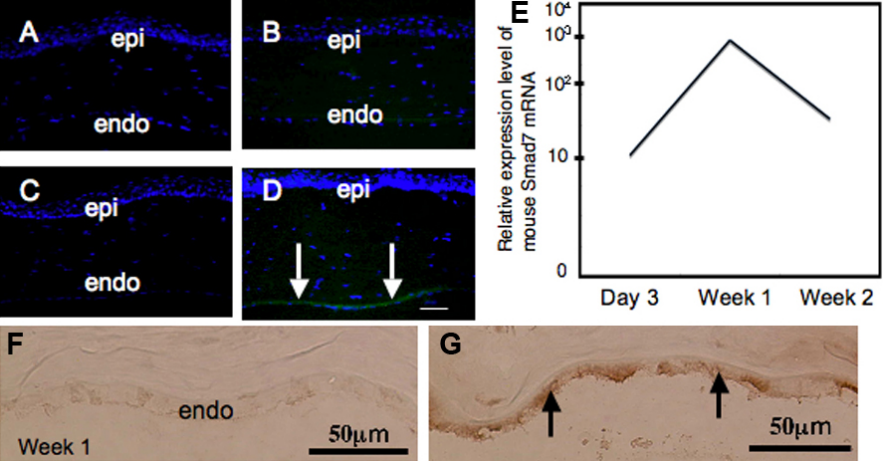
Efficacy of gene transfer by using Cre/LoxP adenoviral vector. A-D: In an unfixed cryosection, exogenous green fluorescein protein (GFP) was observed in the endothelial layer of a cornea treated with Cre-Ad and LNL-GFP-Ad very faintly at day 3 (B) and obviously at week 1 (D, arrows) while it was not seen in the eyes with just Cre-Ad (A,C). Bar, 10 μm. E: Mouse Smad7 mRNA was detected at a high level in Smad7-Ad corneas as early as day 3 and lasted until week 2 but was never detected in the control rat cornea. F-G: Immunohistochemistry showed high intense immunoreaction of Smad7 in the endothelium of a cornea in the Smad7-Ad group (**G**), but this was not seen in a control, Cre-Ad treated cornea (**F**). Bar, 50 μm.

Before examining if the introduction of exogenous mouse *Smad7* cDNA leads to its mRNA expression, we observed the expression of exogenous GFP by using fluorescent microscopy. In an unfixed cryosection, exogenous GFP was observed in the endothelial layer of a cornea treated with Cre-Ad and LNL-GFP-Ad very faintly at day 3 (Figure 5B) and obviously at week 1 (Figure 5D, arrows) while it was not seen in eyes with just Cre-Ad (Figure 5A,C).

Then we examined the expression of *Smad7* mRNA in Smad7-Ad treated corneas by real-time RT–PCR and immunohistochemistry (Figure 5E). Mouse *Smad7* mRNA was detected at a high level in Smad7-Ad corneas as early as day 3 and lasted until week 2 but never in a control rat cornea. Immunohistochemistry showed high, intense immunoreaction of Smad7 at week 1 as shown in Figure 5G.

### Cornea alkali burn model of corneal endothelium fibrosis

**Figure 6:**
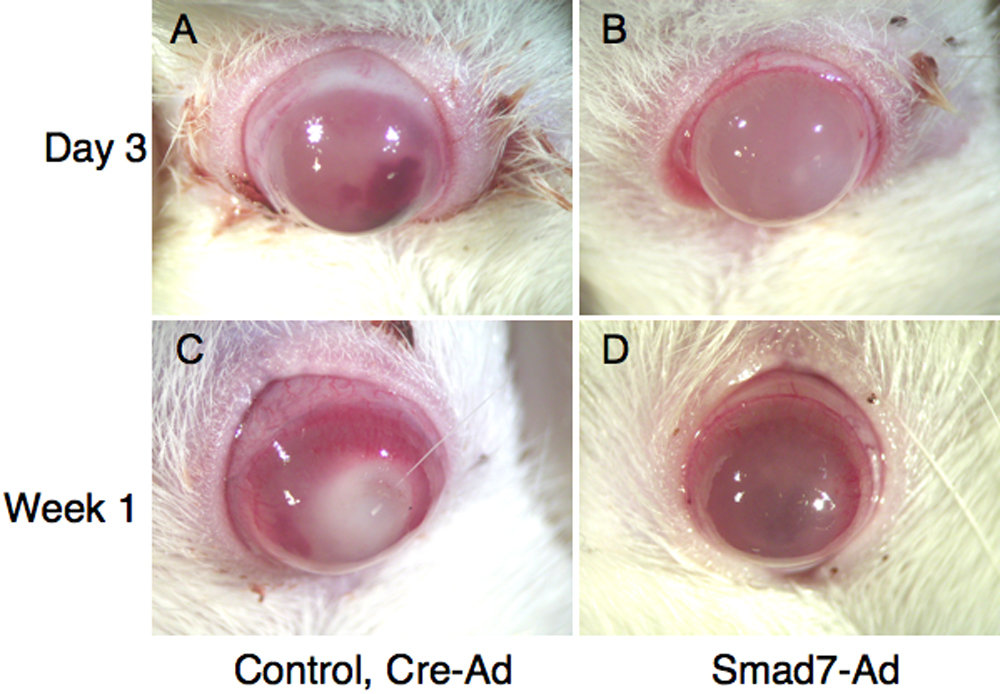
Cornea alkali burn model in rats. Alkali exposure resulted in an epithelial defect, stromal opacity, and hemorrhaging in the anterior chamber. These pathological findings were overall less severe in the Smad7-Ad group (**B**,**D**) as compared with the control Cre-Ad group (**A**,**C**) at day 3 (**A**,**B**) and week 1 (**C**,**D**).

Alkali exposure resulted in an epithelial defect, stromal opacity (inflammation and neovascularization), and inflammation in the anterior chamber as previously reported [25]. However, these pathological findings were overall less severe in the Smad7-Ad group when compared to the control Cre-Ad group at each time point (Figure 6).

**Figure 7:**
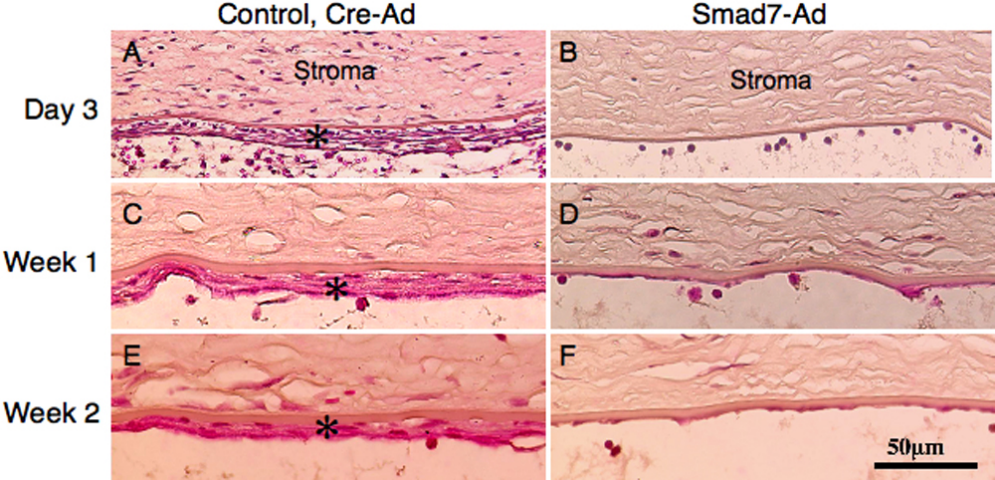
Hematoxylin and eosin staining of the corneal endothelium during healing after alkali exposure. Histology showed that there was abnormal accumulation of fibrous tissue (asterisk) posterior to the Descemet’s membrane at day 3 (**A**), week 1 (**C**), and week 2 (**E**) in the eyes of the control Cre-Ad group while such findings were not observed in the eyes of the Smad7-Ad group at each time point (**B**,**D**,**F**). Inflammation in the stroma was also less severe in a Smad7-Ad treated cornea compared to a control cornea at day 3. Bar, 50 μm.

As for the endothelium, HE staining histology showed that there was abnormal accumulation of fibrous tissue (asterisk) posterior to Descemet’s membrane at day 3, week 1, and week 2 in the eyes of the control Cre-Ad group (Figure 7A,C,E) while such findings were not observed in the Smad7-Ad group eyes at each time point (Figure 7B,D,F).

**Figure 8:**
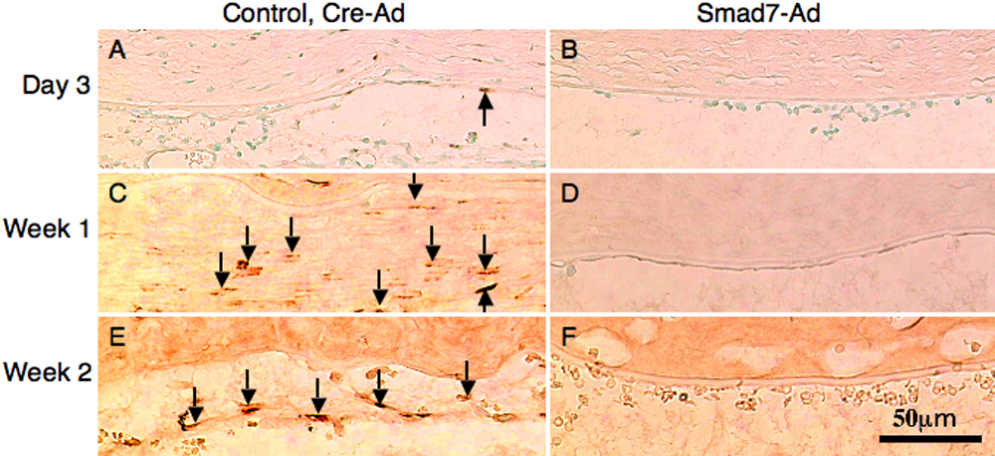
Expression pattern of phospho-Smad2 in healing endothelium of an alkali-burned cornea. Immunohistochemistry showed that the cells in the fibrous tissue in the control eyes were labeled for nuclear phospho-Smad2 at day 3 (**A**) and the number of such labeled cells increased in the fibrous tissue that formed posterior to Descemet’s membrane at week 1 (**C**) and week 2 (**E**). Phopsho-Smad2 positive cells were not seen in the endothelial layer of Smad7-Ad eyes at each time point (**B**,**D**,**F**). The finding indicates that exogenous Smad7 might block Smad signaling. Bar, 50 μm.

**Figure 9:**
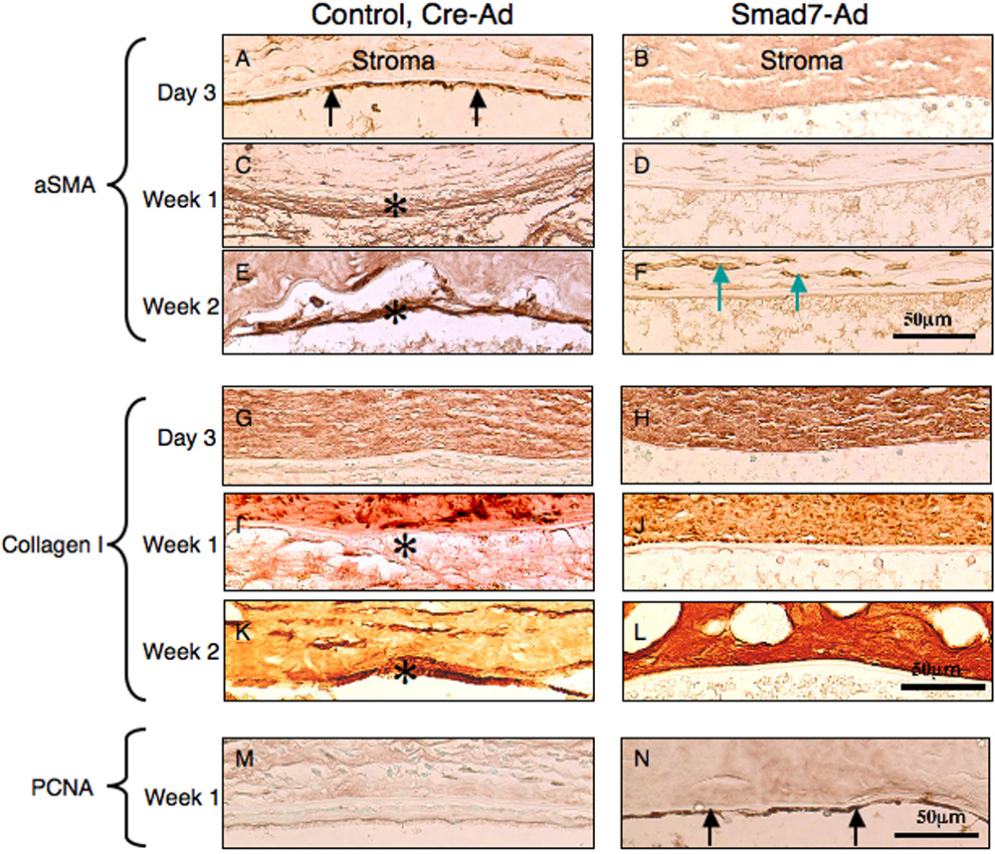
Expression pattern of a marker for endothelial-mesenchymal transition, α-smooth muscle actin, and that for fibrogenic reaction, type I collagen, as well as PCNA in the healing, post-alklai burn, endothelium. The cells in the fibrous tissue posterior to Descemet’s membrane in the control eyes were labeled for αSMA, the marker of myofibroblast or EnMT, as early as day 3 (**A**, arrows; compare to **B**). The cells in such a fibrous structure were markedly labeled at week 1 (**C**) and week 2 (**E**). Such labeled cells were not seen in the corneas of the Smad7-Ad group throughout the intervals of healing. Stromal cells exhibited positive αSMA at week 2 (**F**). Type I collagen was stained as a marker of fibrous matrix accumulation. In the eyes of the control group, type I collagen was detected in the matrix of fibrous tissue formed at week 1 (**F**) and week 2 (**K**) but not at day 3. No type I collagen immunoreactivity was observed posterior to the Descemet’s membrane in the eyes of the Smad7-Ad group throughout the intervals (**H**,**J**,**L**). Nuclear staining of PCNA was more prominent in an eye of the Smad7-Ad group (**N**) than in an eye of the Cre-Ad group (**M**). Such a difference of PCNA staining was not seen between the groups at day 3 or at week 2 (data not shown). Bar, 50 μm.

Immunohistochemistry first showed that the cells in the fibrous tissue in the control eyes were labeled for nuclear phospho-Smad2 at each time point while not seen in eyes of the Smad7-Ad group (Figure 8), indicating that exogenous Smad7 might block Smad signaling. The cells in the fibrous tissue in the control eyes were labeled for αSMA, the marker of myofibroblast or EnMT, while this marker was not seen in the eyes of the Smad7-Ad group (Figure 9). Type I collagen was stained as a marker of fibrous matrix accumulation. Type I collagen was detected in the matrix of fibrous tissue formed in eyes of the control group (Figure 9). Nuclear staining of PCNA was more prominent in an eye of the Smad7-Ad group as compared with an eye of the Cre-Ad group (Figure 9). Such a difference of PCNA staining was not seen between the groups at day 3 or at week 2 (data not shown).

**Figure 10:**
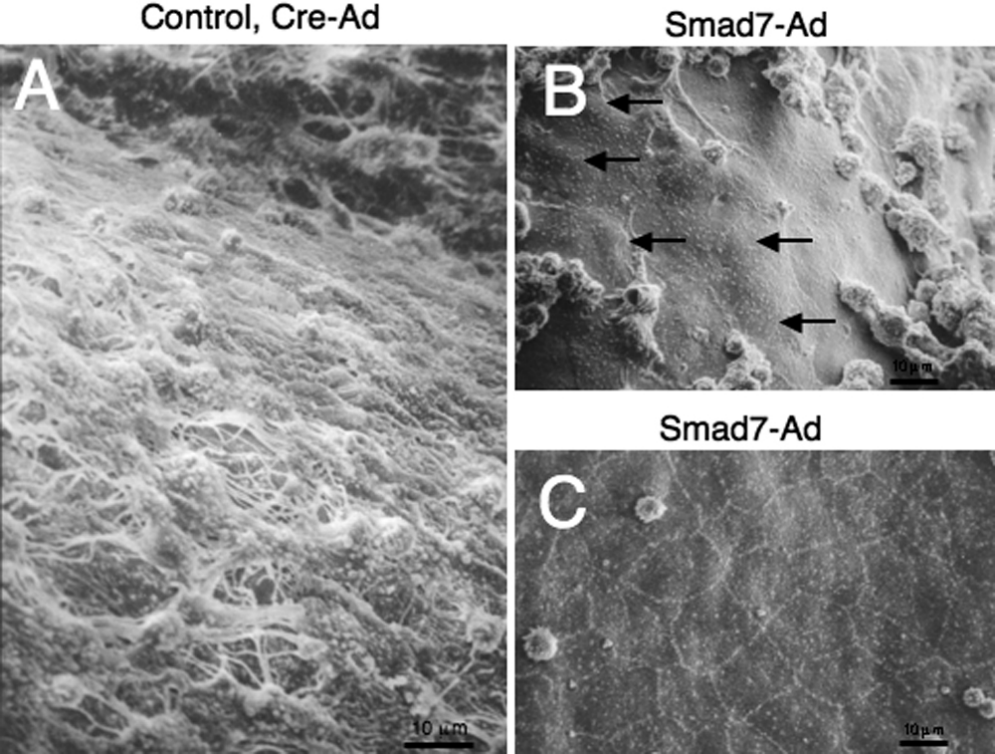
Morphology of the endothelial surface as observed by scanning electron microscopy. Ultrastructurally, the inner surface of the cornea was occupied with elongated cells of a fibroblast-like morphology in an eye of the control group at week 1 (**A**). On the other hand, in an eye of the Smad7-Ad group, the inner corneal surface was found to be covered with enlarged flattened cells with a central nuclear elevation (arrows) in association with adhesion of spheroid cells in the central corneal zone (**B**). The inner corneal surface was also to be covered with relatively normal-like endothelial cells with a clear cell-cell contact, although the cells did not exhibit a hexagonal shape (**C**). Bar, 10 μm.

Scanning electron microscopy showed that the inner surface of the cornea was occupied with elongated cells of a fibroblast-like morphology in an eye of the control group at week 1 (Figure 10A). On the other hand, in an eye of the Smad7-Ad group, the inner corneal surface was found to be covered with enlarged flattened cells with a central nuclear elevation (arrows) in association with adhesion of spheroid cells in the central corneal zone and also to be covered with relatively normal-like endothelial cells with a clear cell-cell contact, although the cells did not exhibit a hexagonal shape (Figure 10B,C).

## Discussion

We first examined the roles of cytokines/growth factors and their signals in the healing of the corneal endothelium in organ culture. In a 24 h culture, the cell spreading of the endothelial sheet toward the defect lacked PCNA staining, and thus, cell migration was the main component of defect repair in this experimental system. We then showed that endothelial repair after producing a defect was accelerated by exogenous EGF, and this acceleration was counteracted by the further addition of exogenous TGF-β1/2. However, endogenous TGF-β is considered to be essential to endothelial migration as the neutralizing antibody retarded the endothelial sheet spreading. Endogenous TGF-β secreted by endothelium or by both the endothelium and keratocytes is considered to be required for endothelium migration in that the neutralizing antibody retarded its migration. We consider that endogenous TGF-β was enough to affect endothelium migration and further addition of exogenous TGF-β1/2 did not do anything. In the present study, EGF was used as one of the major growth factors that reportedly promote cell migration in an injured tissue. However, endogenous TGF-β might not be enough to counteract an EGF-mediated promotion of cell migration. However, this EGF action was considered to be blocked by a large amount of TGF-β1/2 added to the culture medium. We then had to detect the role of each TGF-β related signal in endothelial spreading/migration before we planned an in vivo gene transfer experiment that targets TGF-β driven  EnMT and subsequent fibrogenic reaction in the corneal endothelium. It was required to prevent the inhibition of spreading/migration-promoting signal when we tried to block EnMT by targeting TGF-β related signals. The results showed that the p38 and Erk signals were involved in endothelial defect repair (mainly by cell migration) while Smad and JNK had minimal roles in it. Our previous study showed that myofibroblast generation by epithelial types depend on Smad and/or p38 both in vitro and in vivo [17-19,26]. Smad7 is an inhibitory Smad molecule that blocks TGF-β activated Smad2/3 signal. Injury induced tissue fibrosis in the crystalline lens or fibrosis in the retinal pigment epithelium after producing experimental retinal detachment were prevented by the adenoviral gene transfer of *Smad7* or dominant-negative p38MAPK in mice [17-19,26]. p38 signaling reportedly phosphorylates the middle-linker region of Smad2/3 molecules but not the classical COOH-terminal region of Smad2/3 [27,28]. Our previous experiments showed that p38-mediated phosphorylation of the Smad middle linker region is required for the full activation of Smad dependent gene expression in retinal pigment epithelial cells but not in fibroblasts in vitro [26]. However, our present study clearly showed that the inhibition of p38 is unfavorable for the purpose of promoting endothelial repair. These reports also encouraged us to use *Smad7* gene transfer to promote endothelial healing after an alkali burn by inhibiting myofibroblast generation and fibrogenic reaction in the corneal endothelium.

We therefore tried to suppress an injury-induced, unfavorable fibrogenic reaction of corneal endothelial cells in vivo by *Smad7* gene introduction. We employed the Cre/LoxP system of adenoviral gene transfer of *Smad7* that blocks phosphorylation of TGF-β driven Smad2/3 activation.

Indeed, Smad7 overexpression suppressed an injury-induced fibrogenic reaction of the corneal endothelium in vivo in rats. Expression of αSMA and the accumulation of collagen I were almost completely abolished with Smad7 overexpression. Thus, injury-induced EnMT and subsequent endothelial fibrosis was blocked by Smad7. Phospho-Smad2 labeled cell nuclei were much less observed, which means that overexpressed Smad7 acted to block the Smad signal. We also observed an increase in cell proliferation in the endothelium as detected by PCNA immunoreactivity. Blocking TGF-β/Smad signaling might in turn accelerate cell proliferation. The mechanism of promoting cell proliferation by blocking Smad signaling might be speculated with in vitro studies. It was reported that TGF-β/Smad inhibits epithelial cell growth in part via transcriptional induction of the cell cycle inhibitor, p21 (WAF1/Cip1; p21). A pancreatic cancer cell line engineered to overexpress Smad7 are resistant to the actions of TGF-β1 with respect to growth inhibition and cisplatin-induced apoptosis. Smad7 overexpression interfered with TGF-β1 mediated attenuation of cyclin A and B levels, the inhibition of cdc2 dephosphorylation and cyclin dependent kinase 2 (CDK2) inactivation, upregulation of p27, and the maintenance of the retinoblastoma protein in a hypophosphorylated state [29,30] Moreover, opacification of the corneal stroma also seemed less in the Smad7-Ad group as compared with the control group, suggesting that the promotion of endothelial repair might be beneficial in the restoration of homeostasis in a whole cornea.

It was reported that fibroblast growth factor 2 (FGF-2) promotes EnMT via activation of phosphatydil inositol-3 (PI-3) kinase like other epithelial cell types, i. e., a renal tubular epithelial cell [6,31,32]. Moreover, FGF-2 performs an additional promotion of TGF-β driven EnMT or epithelial-mesenchymal transition (EMT) in a renal tubular epithelial cell or cancer cells. Thus, signaling pathways toward EnMT or EMT originated from TGF-β and FGF-2, and they may be independent of each other.

In conclusion, blocking Smad signaling has a minimal effect on the repair of a defect in the corneal endothelium and also *Smad7* gene transfer effectively suppresses injury-induced fibrogenic reaction in the corneal endothelium. We have previously reported that natural constituents of an herbal medicine exhibit anti-Smad effects in vitro and also therapeutic effects in the treatment of tissue fibrosis in the liver or cornea in vivo. The finding indicates that blocking Smad2/3 signaling irrespective the methods might be effective in the treatment of corneal endothelial fibrosis.
